# Evaluation of the Antioxidant and Neuroprotectant Activities of New Asymmetrical 1,3-Diketones

**DOI:** 10.3390/molecules23081837

**Published:** 2018-07-24

**Authors:** Carla I. Nieto, María Pilar Cornago, María Pilar Cabildo, Dionisia Sanz, Rosa M. Claramunt, María Carmen Torralba, María Rosario Torres, Diana Martínez Casanova, Yaiza Rebeca Sánchez-Alegre, Esther Escudero, José Luis Lavandera

**Affiliations:** 1 Departamento de Química Orgánica y Bio-Orgánica, Facultad de Ciencias, Universidad Nacional de Educación a distancia (UNED), Paseo Senda del Rey 9, E-28040 Madrid, Spain; carla.nieto@hotmail.com (C.I.N.); mcornago@ccia.uned.es (M.P.C.); pcabildo@ccia.uned.es (M.P.C.); dsanz@ccia.uned.es (D.S.); 2 Departamento de Química Inorgánica I and CAI de Difracción de Rayos-X, Facultad de Ciencias Químicas, Universidad Complutense de Madrid (UCM), E-28040 Madrid, Spain; torralba@ucm.es (M.C.T.); mrtorres@ucm.es (M.R.T.); 3 Instituto de Medicina Molecular Aplicada (IMMA), Facultad de Medicina, Universidad CEU San Pablo, Campus de Montepríncipe, Boadilla, E-28668 Madrid, Spain; d.martinez38@usp.ceu.es (D.M.C.); yaizarebeca.sanchezalegre@hotmail.com (Y.R.S.-A.); estheresc@ceu.es (E.E.)

**Keywords:** neurodegeneration, oxidative stress, neuroprotectant, antioxidant, β-diketones, drug-like properties

## Abstract

A series of fourteen new asymmetrical 1,3-diketone derivatives have been synthesized and evaluated in the ABTS, FRAP and DPPH assays as a new chemotype with antioxidant and drug-like properties. All the compounds displayed low cytotoxicity in comparison to curcumin against the human neuroblastoma SH-SY5Y cell line. Among them, (3*Z*,5*E*)-6-(2,5-difluoro-4-hydroxy-phenyl)-1,1,1-trifluoro-4-hydroxyhexa-3,5-dien-2-one (**6b**) and (3*Z*,5*E*)-6-(2,3-difluoro-4-hydroxy-phenyl)-1,1,1-trifluoro-4-hydroxyhexa-3,5-dien-2-one (**7b**) with excellent solubility and chemical stability in biorelevant media, have also shown a similar Fe^+2^ chelation behavior to that of curcumin. Additionally, both derivatives **6b** and **7b** have afforded good neuroprotection activity against H_2_O_2_ induced oxidative stress in the same neuronal cell line, with a significant reduction of intracellular ROS levels, in parallel with a good recovery of the Mitochondrial Membrane Potential (ΔΨ_m_). Compounds **6b** and **7b** with a promising antioxidant and drug-like profile, with low cytotoxic and good neuroprotectant activity, constitute a new interesting chemical class with high potential as new therapeutic agents against neurodegenerative diseases.

## 1. Introduction

Neurodegenerative diseases are a diverse group of pathologies of the nervous system that have many different aetiologies and represent an important social and financial burden on the society. Their prevalence is growing, mainly due to the increase in life expectancy. According to a 2015 United Nations report on world population ageing, the number of people aged 60 and older worldwide will reach almost 2.1 billion people in the next 35 years [1]. Despite this improvement in life span, aging is the cause of neurodegeneration [2], a gradual deterioration of brain and nerves, with pathological abnormalities of relatively specific populations of neurons and specific regions of the brain [3], with characteristic changes that include a loss of cognitive and motor abilities [3,4]. Neurodegeneration is the common feature of a wide number of diseases that include Alzheimer’s disease (AD), Parkinson’s disease (PD), amyotrophic lateral sclerosis (ALS), Huntington disease and Friedreich Ataxia, for which currently, there are no therapies available to cure any of these diseases, and there is only treatment to alleviate the symptoms [5,6,7,8,9].

Although a small proportion (less than 5%) of these neurodegenerative diseases have a genetic origin, most of them (sporadic diseases) have an unknown origin that could be secondary to metabolic or toxic mechanisms [10,11,12,13], that lead to mitochondrial dysfunction [14,15,16,17,18,19] and molecular processes that build up neurotoxic protein aggregates [20,21,22,23,24,25,26]. Most of the actual research focuses on the similarities in neurodegeneration that occur in each of these diseases [26,27,28,29,30,31,32]. Through identifying these parallels, researchers hope to understand the mechanisms of disease in order to improve their chances of developing new therapies. Among other mechanisms, oxidative stress (OS) has been long recognized as a key player in the pathogenesis of neurodegenerative diseases [9,33,34,35,36,37,38,39,40,41,42]. The brain is a metabolically hyperactive organ with less capacity for the cellular regeneration compared to other organs and is extremely vulnerable to oxidative damage due to high oxygen consumption and low antioxidant defense, and an abundance of oxidation-sensitive lipids. Generation of free radicals, mainly reactive oxygen species (ROS) and reactive nitrogen species (RNS), is an integral part of normal cellular function like the mitochondrial respiratory chain and is generally thought to be the main cause of oxidative stress [33,35,43]. ROS/RNS at moderate concentrations have important roles in physiological processes and signaling pathways. Oxidative stress occurs when there is an excessive formation of ROS/RNS overwhelms the cell antioxidant mechanisms, which are maintained by antioxidant enzymes which include superoxide dismutase (SOD), catalase and glutathione peroxidase, and endogenous antioxidants such as ascorbic acid, alpha-tocopherol, glutathione (GSH), carotenoids and flavonoids. Failure of endogenous antioxidant defenses leads to oxidative damage of proteins, lipids and DNA/RNA, which are the common features of many neurodegenerative diseases [35,43].

There are many different ROS being the non-radical hydrogen peroxide (H_2_O_2_), the superoxide radical (O_2_^•*−*^) and the hydroxyl radical (OH^•^) the most important ones [9,33,43], which are produced either by enzyme or metal-catalyzed processes or in the mitochondrial electron transport chain, during energy transduction [17,18,44,45].

According to the facts that oxidative stress is pathogenic in neurodegenerative diseases, there is a rationale to use antioxidants as potential therapies [46,47,48,49,50,51,52,53]. A number of studies have shown beneficial effects of antioxidants in animal and cell models of neurodegenerative disease in cell culture toxicity studies. The most widely antioxidant therapies studied of these have been vitamin E, vitamin C, coenzyme Q10, creatine, melatonin, *N*-acetylcysteine, curcumin and other phenolic and flavonoid compounds [33,54,55,56].

**Figure 1:**
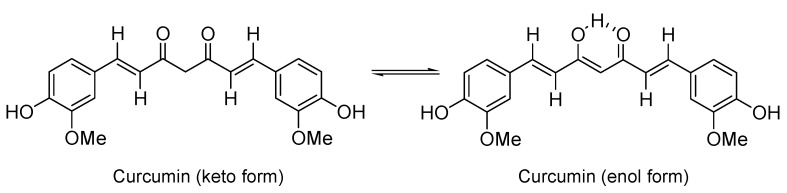
Curcumin represented in its two possible tautomeric forms.

Curcumin (Figure 1) and its close derivatives are polyphenol compounds from turmeric (*Curcuma longa*), that have shown diverse biological properties that modulate biochemical processes involved in AD [57,58,59] that include attenuation of mitochondrial dysfunction-induced oxidative stress and inflammatory responses to inflammatory cytokines, COX-2, and iNOS. Curcumin also binds to β-amyloid (Aβ) plaques to inhibit amyloid accumulation and aggregation, and the toxic Aβ oligomer formation, avoiding oligomer-dependent Aβ toxicity in the brain [60,61,62,63]. This molecule also prevents α-synuclein aggregation in PD and attenuate ROS-induced COX-2 expression in ALS [64,65]. These results demonstrate that curcuminoids may be effective therapeutic tools to treat neurodegenerative diseases. Nevertheless, due to curcumin low water solubility and poor absorption after oral administration, there are several pharmacokinetic and pharmacodynamic issues such as its low bioavailability and metabolic stability that hinder the effective targeting of this molecule to the cells and mitochondria in human brain [66,67,68,69,70]. Despite the promise of curcumin in animal and in vitro cell studies, there has been no proven benefit for the use of this molecule in either AD or PD from large randomized controlled trials [71,72,73].

Notwithstanding, curcuminoids are promising chemotypes amenable for chemical modification that can overcome those drug-like issues. Extensive synthetic work has been focused on the development of new curcuminoid derivatives with a similar antioxidant profile as curcumin mainly affording symmetric derivatives but with different aromatic substitution patterns [74,75].

**Figure 2:**
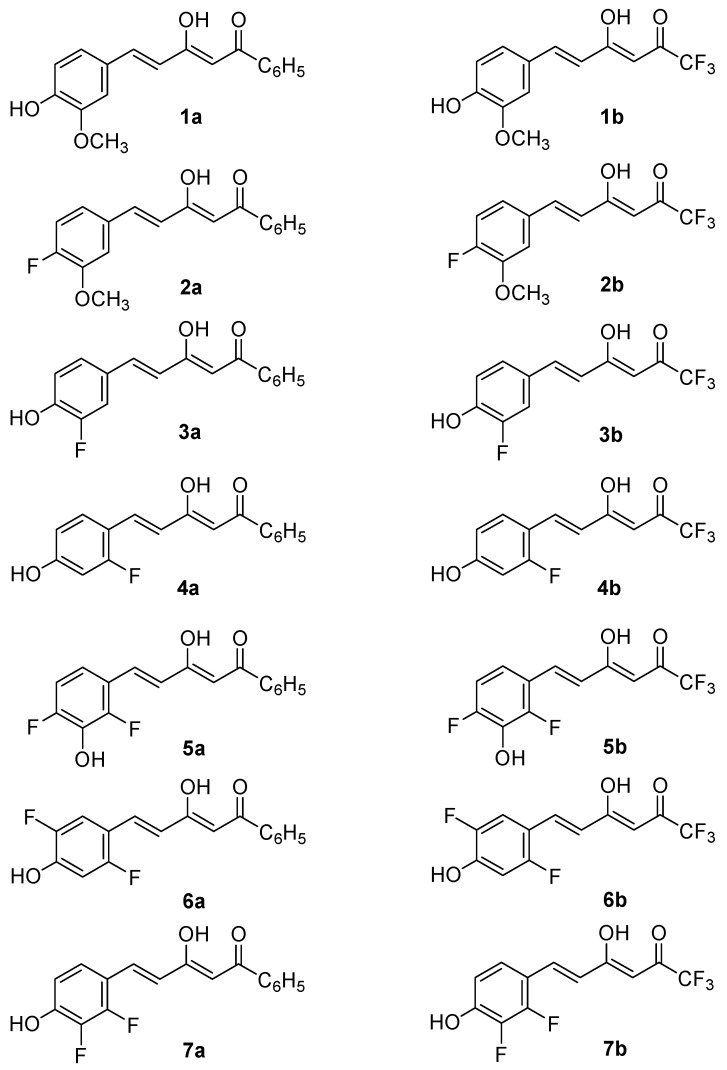
Curcuminoids studied in this paper, represented as the major tautomeric form.

Taking the above into account, the present work reports the synthesis and biological evaluation of new asymmetric curcuminoid derivatives with antioxidant and in vitro cell neuroprotective properties, and improved drug-like profile, as potential starting points for the development of new neuroprotectants. The two series of compounds approached are the hemicurcuminoids β-diketones, reported in Figure 2.

## 2. Results and Discussion

### 2.1. Synthesis and Structure Characterization

**Scheme 1:**
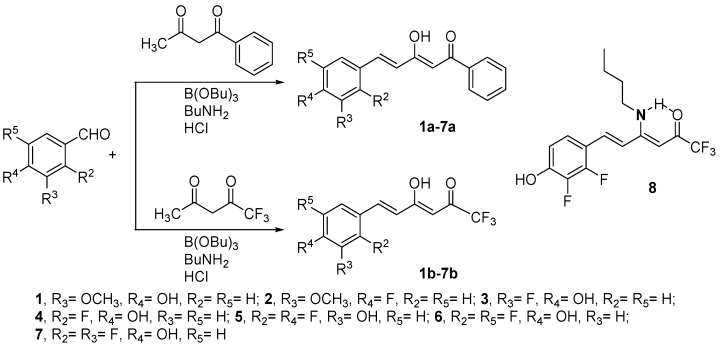
Synthetic scheme used to prepare compounds **1a**–**7a** and **1b**–**7b**.

With the exception of curcumin which is commercially available and has been used after purification by crystallization from ethanol-water, the preparation of the studied β-diketones was achieved by the Pabon method [76] starting from the conveniently substituted benzaldehyde and reacting it with 1-phenylbutane-1,3-dione to yield compounds **1a**–**7a,** or with 1,1,1-trifluoropentane-2,4-dione to get compounds **1b**–**7b** (Scheme 1). In the case of compound **7b** it was found necessary to use 12 M instead of 1 M HCl, to completely hydrolyze the formed enamino-ketone **8** (see Section 4). As the β-diketones are not symmetrical they can exist in two enol tautomeric forms, the most stable one being reported in Figure 2. Consequently, we have named them applying the IUPAC rules to the major keto-enol form.

Compounds **7a** and **7b** are described here for the first time, all others were previously prepared and their structural characterization already reported by us [77,78,79]. The NMR spectra confirm in both cases that the predominant tautomer corresponds to the enol close to the styryl group. In (2*Z*,4*E*)-5-(2,3-difluoro-4-hydroxyphenyl)-3-hydroxy-1-phenylpenta-2,4-dien-1-one (**7a**), the chemical shift of the OH is observed at 16.26 ppm (DMSO-*d*_6_) and in (3*Z*,5*E*)-6-(2,3-difluoro-4-hydroxyphenyl)-1,1,1-trifluoro-4-hydroxyhexa-3,5-dien-2-one (**7b**) at 14.15 ppm (CDCl_3_). In ^13^C-NMR the most representative values confirming it are: **7a**, C_6_H_5_**C**O-, 188.5 (CPMAS, 192.1), =**C**HCO, 97.8 (CPMAS, 98.2) and CH=**C**(OH), 179.3 (CPMAS, 176.1) ppm and **7b**, CF_3_**C**O-, 179.3, =**C**HCO, 95.0 and CH=**C**(OH), 180.6 ppm [79,80].

**Figure 3:**
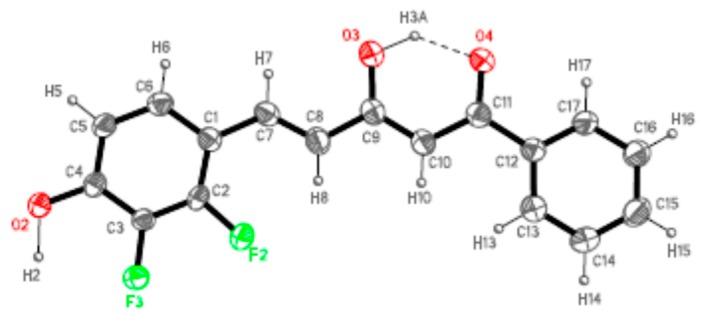
ORTEP plot (30% probability for the ellipsoids) of **7a** showing the labelling of the asymmetric unit.

**Figure 4:**
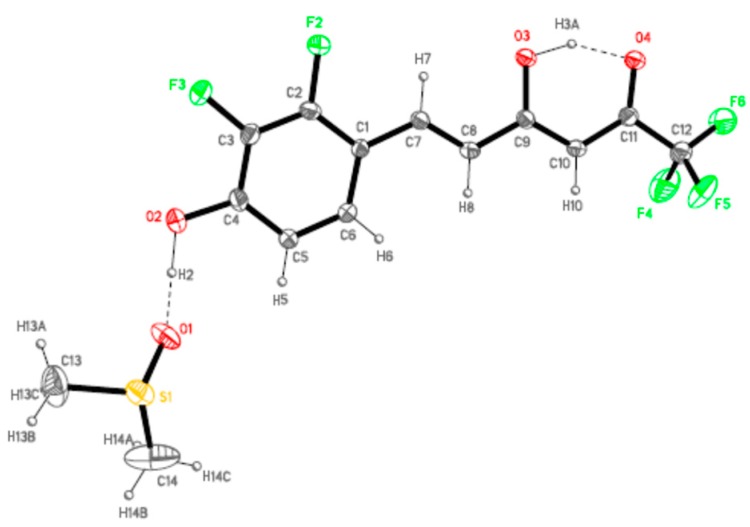
ORTEP plot (20% probability for the ellipsoids) of **7b** showing the labelling of the asymmetric unit.

Following, as suitable single crystals for X-ray diffraction studies were obtained for **7a** from chloroform-methanol and for **7b** from hexane/acetyl acetate, the molecular and crystal structure investigation was undertaken. Compounds **7a** and **7b** crystallize in *Pna*2_1_ and *Pbca* orthorhombic space groups, respectively. Figure 3 and Figure 4 display the ORTEP plot [81] together with the representative labeling scheme of the asymmetric unit and Table 1 summarizes the hydrogen bond data.

Both derivatives present one single molecule per asymmetric unit, with one DMSO crystallization molecule in compound **7b**. As expected, the molecules of these derivatives are almost planar in agreement with an extended electronic delocalization deduced from the bond distances values. The maximum dihedral angle between the phenol ring and the rest of the molecule is 3.9(5)° for **7a** and 6.6(3)° for **7b**.

In accordance with our previous results [78,79], all the molecules are in the keto-enol form observed by NMR (see Figure 2), the C11–O4 bonds [1.253(3) and 1.241(6) Å] show higher order than the C9–O3 ones [1.329(3) and 1.328(6) Å] for **7a** and **7b**, respectively. Another common feature to point up is the formation of a strong intramolecular hydrogen bond, O3H3A···O4, with different strength in both derivatives but with an analogous distance O3···O4 indicating a similar symmetrization of this moiety (Table 1). This fact is related to the different intermolecular interactions observed in both derivatives.

**Figure 5:**
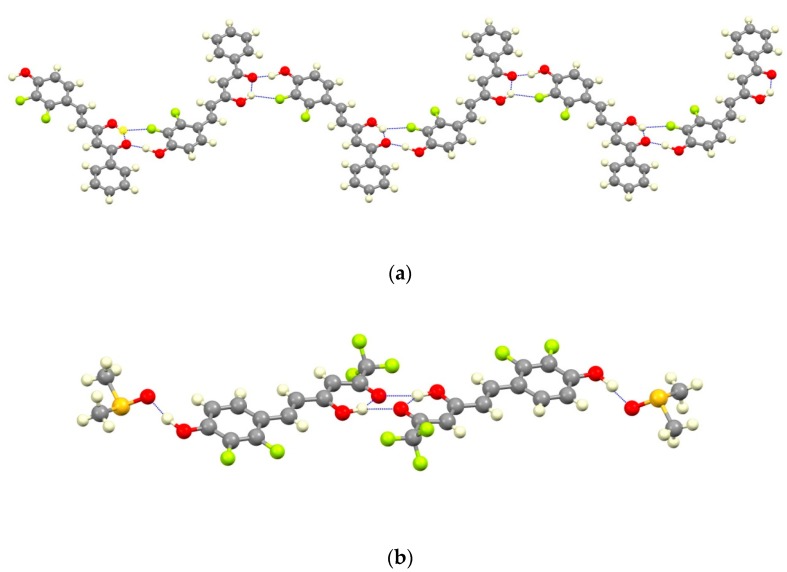
View of a chain and the dimer formed by different H-bonding interactions for **7a** (**a**) and **7b** (**b**).

For **7a** two different intermolecular interactions can be described, a strong hydrogen bond between the phenolic group and the ketonic oxygen (O2H2···O4#1; Table 1) and, an additional weaker interaction between the enol hydrogen and the F3 atom in *ortho* position relative to the phenolic group (distance O3H3A···F3#1 of 2.264(6) Å). These intermolecular hydrogen bonds between adjacent coplanar molecules lead to the formation of zig-zagged chains as shown in Figure 5; there are no significant additional interactions between adjacent chains.

**Figure 6:**
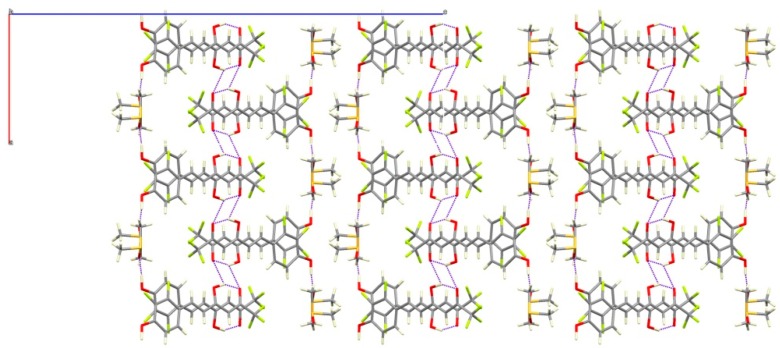
View of the crystal packing of **7b** along *b*-axis.

The presence of DMSO molecules in compound **7b** determines the intermolecular interactions. In that way, the phenolic group of **7b** forms a hydrogen bond with the oxygen of the DMSO molecule, preventing the interaction with the ketonic oxygen O4 found in **7a**. For that reason, the ketonic groups of two adjacent molecules are oriented to each other giving rise to the formation of a double hydrogen bond [distances O3H3A···O4#1 of 2.652(5) and O3···O4#1 3.158(5) Å; angle O3H3AO4 of 107.32(3)°] leading to isolated dimers (Figure 5). The sum of these interactions affords a crystal packing where the molecular dimers are alternating with layers of DMSO molecules (Figure 6). 

### 2.2. Antioxidant Properties

In order to characterize the antioxidant and antiradical capacity of new compounds, based on their physicochemical properties, a number of apparently similar analytical methods are available in the literature. These methods can be broadly classified under two main types, i.e., H atom donating ability (HAT) and electron transfer (ET) from antioxidant compounds, where both result in the neutralization of free radicals [82,83]. HAT assays are based on a competitive reaction where antioxidant and substrate compete for radicals, being the 2,2-diphenyl-1-picrylhydrazyl hydrate (DPPH) method one of the most used. ET methods measure the radical reductive capacity of an antioxidant, in a reaction that involves a colorimetric change when reduction takes place. Among ET assays, the 2,2′-azino-bis-3-ethylbenzthiazoline-6-sulphonic acid (ABTS) [84,85] and Ferric Reducing Antioxidant Power (FRAP) [86,87] methods have been the most cited. 

The antioxidant activity of the compounds of the present work has been characterized by the ABTS, FRAP and DPPH methods. The results are shown in Table 2 and Table 3 as IC_50_ values, defined as the amount of antioxidant needed to decrease the initial radical concentration by 50%, and Trolox Equivalent Antioxidant Capacity (TEAC) values defined as the micromolar concentration of a trolox solution having the same antioxidant capacity as a 100 µM solution of the substance. All the results were compared to those obtained for Trolox and curcumin used as reference compounds. 

From Table 2, it can be concluded, that substitution of the allyl group by phenyl (Ph, compounds **1a**–**7a**) or trifluoromethyl (CF_3_, compounds **1b**–**7b**) on the right side of the curcumin molecule, results in derivatives **1a**, **3a**, **6a**, **7a**, **1b**, **7b** with IC_50_s in the ABTS electron transfer assay, similar or slightly higher than that of curcumin, having in general the series with a Ph group, better inhibitory activity of the radical ABTS**^·^**^+^ in comparison with the series with CF_3_ substitution.

Similarly, in the FRAP assay in which the antioxidant character is evaluated as the reducing capacity of Fe^+3^ to Fe^+2^ a similar trend was observed to that from the ABTS test, being the compounds with greater reductive power in the order curcumin > **1a** ≈ **1b** > **3a** ≈ **3b** > **6a** ≈ **7a** > **6b** ≈ **7b**.

The substitution of the *para*-OH group by fluorine in derivatives **2a** and **2b** results in the loss of all antioxidant activity compared to derivatives **1a** and **1b,** in both the ABTS and FRAP assays, which highlights the need of a phenolic OH as it has been reported previously [88,89]. On the other hand, the substitution of the OMe group at position 3 by fluorine in the derivatives **3a** and **3b** produces a slight decrease of the AOX capacity by electron transfer in the ABTS assay compared to the corresponding derivatives **1a** and **1b**, being that activity similar in both the Ph and CF_3_ series. In the same way, compounds **4a** and **4b** in which the fluorine atom has been introduced at the position 2 of the aromatic ring, the loss of AOX character is greater in comparison with its analogs **3a** and **3b**.

The introduction of a second fluorine atom at positions 5 and 2 of the aromatic ring in derivatives **4a** and **4b** resulted in compounds **6a**, **6b** and **7a**, **7b** respectively, which have shown a slight improvement in antioxidant activity by electron transfer in the ABTS assay as well as reducing capacity of Fe^+3^ to Fe^+2^ in the FRAP test. Finally, permutation of the OH group in position 4 to the *meta* position in derivatives **7a** and **7b**, resulted in derivatives **5a** and **5b**, with almost total loss of AOX activity in both assays.

**Figure 7:**
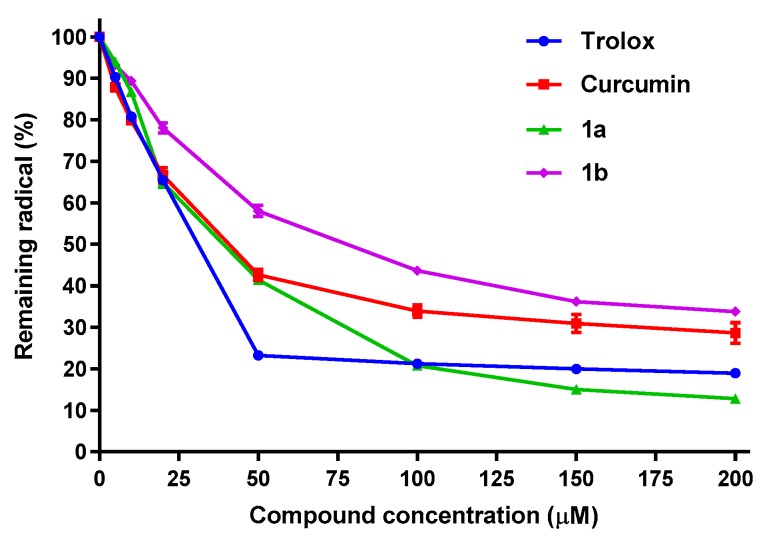
Antioxidant capacity of trolox, curcumin, **1a** and **1b**, measured as % of remaining DPPH^•^ radical. Values are the Mean ± SD of four different experiments carried out by quadruplicate.

Regarding the DPPH assay, which measures the AOX capacity of compounds capable of donating a hydrogen atom or an electron to the DPPH^•^ radical [90,91], compounds **1a** and **1b** were the only β-diketones that have shown activity in this assay. Table 3 displays the IC_50_ values obtained for trolox, curcumin and compounds **1a** and **1b,** while Figure 7 presents also the percent of remaining DPPH^•^ radical vs. compound concentration. Derivatives **1a** and **1b** have slight lower potencies (greater IC_50_ values) than those from curcumin and trolox. The fact that only **1a** and **1b** have DPPH reductive capacity was expected, considering that they keep the methoxy group in alpha position to the phenolic OH. In addition to that, the lack of DPPH activity observed for the fluorinated derivatives, confirms an acidity increase of the phenolic group, produced by the introduction of fluorine atoms in the aromatic ring, which makes the phenolic hydrogen less labile, in agreement with previous results [92].

### 2.3. Calculation of Physicochemical Properties

To study the drug-like potential of all the compounds synthesized in this work, their physicochemical parameters have been calculated and compared to those for curcumin (Table 4). From these data we can conclude that all the compounds have acceptable drug-like properties, with molecular weight (MW) values lower than that of curcumin, with reasonable lipophilicity (clogP) and good predicted clogD (distribution coefficient at pH 7.4), in accordance with the currently most accepted criteria for compounds targeting central nervous system (CNS) i.e., MW ≤ 360, clogP ≤ 3.0, cLogD ≤ 2, HBD ≤ 2, N-Arom-Ring ≤ 2 [93,94]. It is also worth mentioning, the improvement seen in those physicochemical parameters after the replacement of the phenyl ring with a CF_3_ group within the β-diketone chemical series.

On the other hand, Total Polar Surface Area (TPSA) has been used as a predictor for blood-brain barrier (BBB) penetration capacity of drugs [95], where drugs aimed at the CNS tend to have lower polar surface areas. TPSA for a molecule to penetrate the brain has to be >40 Å^2^ and ≤90 Å^2^, in agreement with the most accepted range nowadays [93,94]. From Table 4, we can conclude that the β-diketones have better TPSA in comparison to curcumin, having the CF_3_ derivatives the most compensated profile in MW, clogP, clogD and TPSA, and being compounds **6b** and **7b** the most promising ones, considering their calculated physicochemical properties and antioxidant activities.

### 2.4. Cytotoxicity Studies

SH-SY5Y is a human-derived neuroblastoma cell line well validated as an in vitro neuronal cell line, that has been widely used as an in vitro model for Alzheimer’s and Parkinson’s disease studies [97,98]. On the other hand, Oxidative Stress (OS) is associated with an imbalance between anti-oxidant defenses and pro-oxidant insults which deregulates the homeostatic levels of H_2_O_2_, superoxide radical (O_2_^−^) and hydroxyl radical (OH^•^). In the present work, we hypothesized that asymmetric β-diketones with suitable AOX and drug-like properties could protect SH-SY5Y cells against OS. 

So, to study the neuroprotectant potential of β-diketones in the SH-SY5Y cell line, we firstly need to determine their cytotoxicity in the same cellular model. Table 5 shows the cytotoxicity results in the neuronal cell line SH-SY5Y obtained for compounds reported in this work, expressed as IC_50_ values (µM) i.e., the compound concentration needed to reduce the cell viability to 50% from the control. From these data, we can infer that as a general trend, the replacement of the phenyl group by CF_3_ in β-diketones afforded less cytotoxic derivatives. In addition to that, within each series, the introduction of a second fluorine atom in the left-hand side phenyl ring decreases also the cytotoxicity. We can also conclude that most of the new compounds have shown lower cytotoxicity in this cell line that curcumin, being **6b** and **7b** the most interesting derivatives to test in neuroprotection assays.

### 2.5. Determination of Kinetic Solubility

Among the most important physicochemical parameters in drug-discovery, solubility and chemical stability in aqueous media are the determinants for non-toxic and brain-permeable drug-like compounds with good bioavailability. So, compounds with good aqueous solubility are the best candidates for drug-discovery progression. In order to know the solubility of the most promising β-diketones, **6b** and **7b,** we have determined their kinetic solubility in aqueous media following the procedure described by Hoelke et al. [99]. The assays were performed at room temperature, in 10 mM Phosphate Buffered Saline (PBS) from compound stocks in DMSO at 20 mM concentration. The maximum solubility achieved for compounds **6b**, **7b** and curcumin in 10 mM PBS is shown in Table 6.

It is also important to know compound integrity at those concentrations used in biological assays, and for that, the chemical stability was determined in 10 mM PBS after 6 h, 24 h and 48 h. Results obtained for compounds **6b**, **7b** (at 400 µM) and curcumin (at 50 µM) are shown in Table 7. From these data, we can conclude that derivatives **6b** and **7b** are more stable in aqueous buffer solution than curcumin, which reinforces the drug-like properties of the asymmetrical β-diketone chemical series.

### 2.6. Iron Chelation Activity

Numerous studies have reported that iron induces OS in the degenerative brain and its toxicity might be dependent on the normal function of iron-containing molecules [100,101,102]. In age-related diseases, the dysregulation of iron secretion, transport and storage can result in an imbalance in the normal iron homeostasis and levels of Fe^2+^, which in turn may produce free radicals and OS that overwhelmed endogenous neuronal defenses and generate neurotoxic responses [101]. Iron reacts with H_2_O_2_ in the Fenton’s reaction to produce the highly toxic hydroxyl radical (^•^OH). Also, iron promotes production of ROS that damage lipids, proteins and DNA [103], where iron-induced lipid peroxidation triggers protein aggregation and accumulation [104,105].

**Figure 8:**
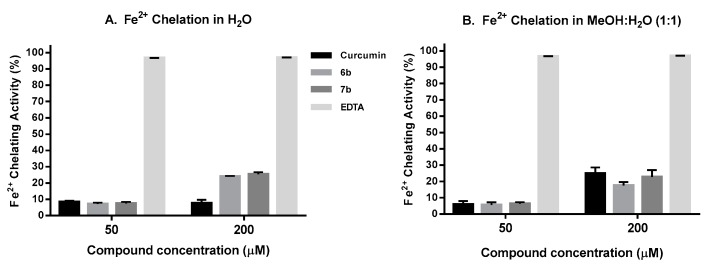
Fe^2+^ Chelating assay results: (**A**) in H_2_O; (**B**) in MeOH:H_2_O (1:1). Data are the Mean ± SD of three independent experiments carried out by quadruplicate *p* < 0.001.

To decrease the level of free Fe^2+^ that can reduce H_2_O_2_ to the ^•^OH radical and produce other ROS, it is worth to evaluate the iron chelation capacity of new potential AOX compounds as potential new therapeutic agents against neurodegenerative diseases [106,107]. In the present work, the Dini’s method [108] was used to evaluate the Fe^+2^ chelating capacity of compounds **6b** and **7b** and curcumin, using EDTA as assay control. In this assay ferrozine form complexes with Fe^+2^ in a quantitative manner, and in the presence of chelating compounds this complex is disrupted. Figure 8A shows the results obtained when the assay was run in aqueous media. At lower compound concentration (50 µM), **6b** and **7b** show similar Fe^2+^ binding capacity (7–8%) to that of curcumin (8%), while at higher concentrations (200 µM) their chelating capacity (25–26%) is higher than that of curcumin, mainly due to their better solubility in aqueous media. When the assay was carried out in a mixture MeOH:H_2_O (1:1), (Figure 8B), the three compounds show a similar Fe^+2^ binding pattern at 50 and 200 µM, where curcumin has shown similar chelating capacity as that previously reported by Dairam et al. [109]. In all the experiments, EDTA completely inhibits the Fe^+2^-Ferrozine complex at both 50 and 200 µM. In summary, compounds **6b** and **7b** have better iron chelation capacity than curcumin in aqueous media, being this feature interesting for potential intracellular Fe^+2^ chelation.

### 2.7. Neuroprotection Studies

To test our work hypothesis, neuroprotection studies were carried out with β-diketones **6b** and **7b** in the SH-SY5Y cell line under oxidative stress conditions. Cells treatment with H_2_O_2_ as stressor is a good in vitro model to evaluate the potential neuroprotective character on new AOX compounds. In the present work after cell treatment with compounds for 24 h, cells were exposed to H_2_O_2_ lethal dose (EC_50_).

**Figure 9:**
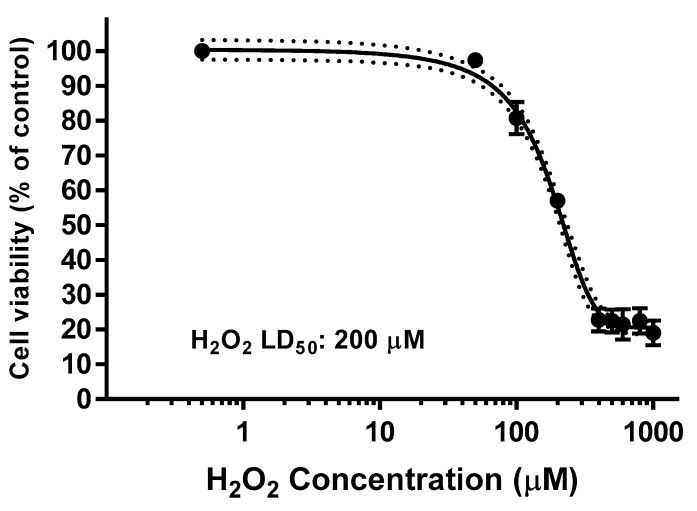
Determination of H_2_O_2_ lethal dose (LD_50_, µM) after 24 h incubation for SH-SY5Y cells. Values are the Mean ± SD of six different experiments carried out by quadruplicate (r^2^: 0.9896).

The H_2_O_2_ EC_50_ in SH-SY5Y cell line was previously determined from the killing H_2_O_2_ curve as it is shown in Figure 9, being 200 µM the concentration for which the cell viability remains as 50% of the control.

**Figure 10:**
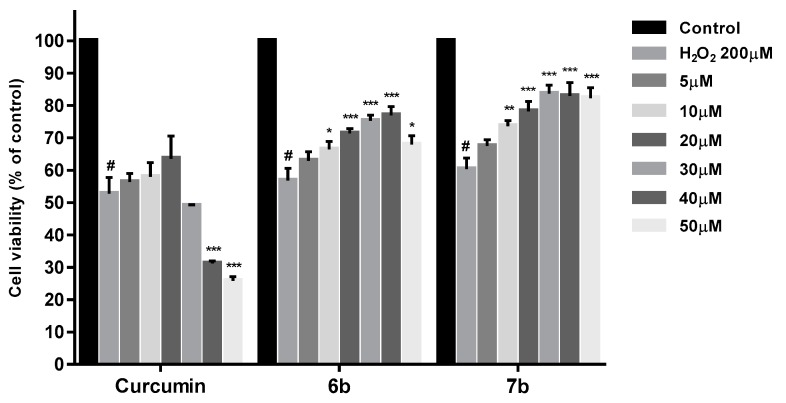
Neuroprotection effect of curcumin, **6b** and **7b** against H_2_O_2_ induced cytotoxicity in SH-SY5Y cells. Cell viabilities were obtained by MTT assay. Compounds were administered at different concentrations for 24 h prior to H_2_O_2_ 200 µM treatment for other 24 h. Data are presented as Mean ± SD of four independent experiments carried out by quadruplicate. ^#^ *p* < 0.001 vs. vehicle-treated control. * *p* < 0.05, ** *p* < 0.01, *** *p* < 0.001 vs. only H_2_O_2_ treated cells.

For neuroprotection experiments, SH-SY5Y cells were treated with test compound at different concentrations (5, 10, 20, 30, 40, and 50 µM) for 24 h. After the incubation period, cells were exposed to H_2_O_2_ 200 µM for 24 h. At the end of the treatment, cell viability was measure with MTT. Figure 10 displays the cell viability results (%) obtained for compounds **6b**, **7b** and curcumin in comparison with control cells. As shown, the decreased SH-SY5Y cell viability cause by 200 µM H_2_O_2_ was significantly improved by pretreatment with **6b** and **7b** at all the concentrations assayed, having **7b** the best cell death inhibition profile at 10 (*p* < 0.01), 20, 30, 40 and 50 µM (*p* < 0.001). However, in the same assay, curcumin has only shown a slight protection at 5, 10 and 20 µM. 

**Figure 11:**
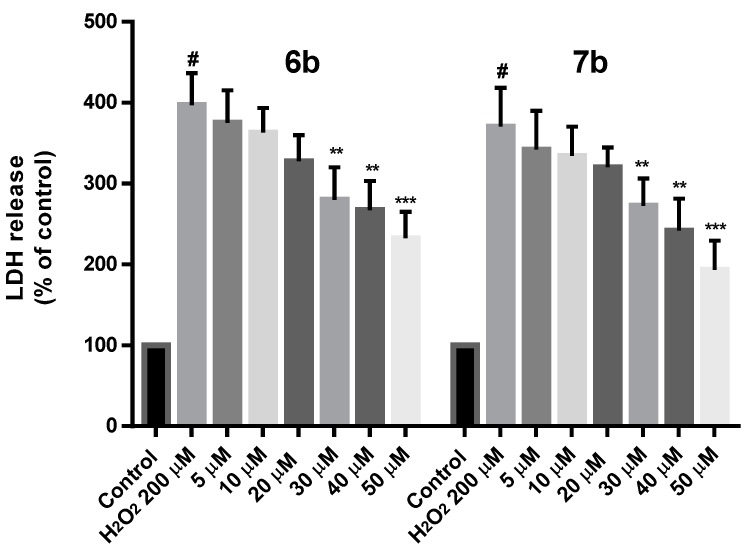
Neuroprotection effect of **6b** and **7b** against H_2_O_2_ induced cytotoxicity in SH-SY5Y cells expressed as LDH release. Compounds were administered at different concentrations for 24 h prior to H_2_O_2_ 200 µM treatment for 24 h. Data are presented as Mean ± SD of three independent experiments carried out by quadruplicate. ^#^ *p* < 0.001 vs. vehicle-treated control. * *p* < 0.05, ** *p* < 0.01, *** *p* < 0.001 vs. only H_2_O_2_ treated cells.

Additionally, the protection effect of **6b** and **7b** on cell death induced by 200 µM H_2_O_2_ was determined by measuring the lactate dehydrogenase (LDH) release in cell culture medium. As shown in Figure 11, both **6b** and **7b** reduced the LDH leakage at all the concentrations tested, being this effect more significant at 30, 40 (*p* < 0.01) and 50 µM (*p* < 0.001).

### 2.8. Effect on ROS Levels in SH-SY5Y Cells under Oxidative Stress

**Figure 12:**
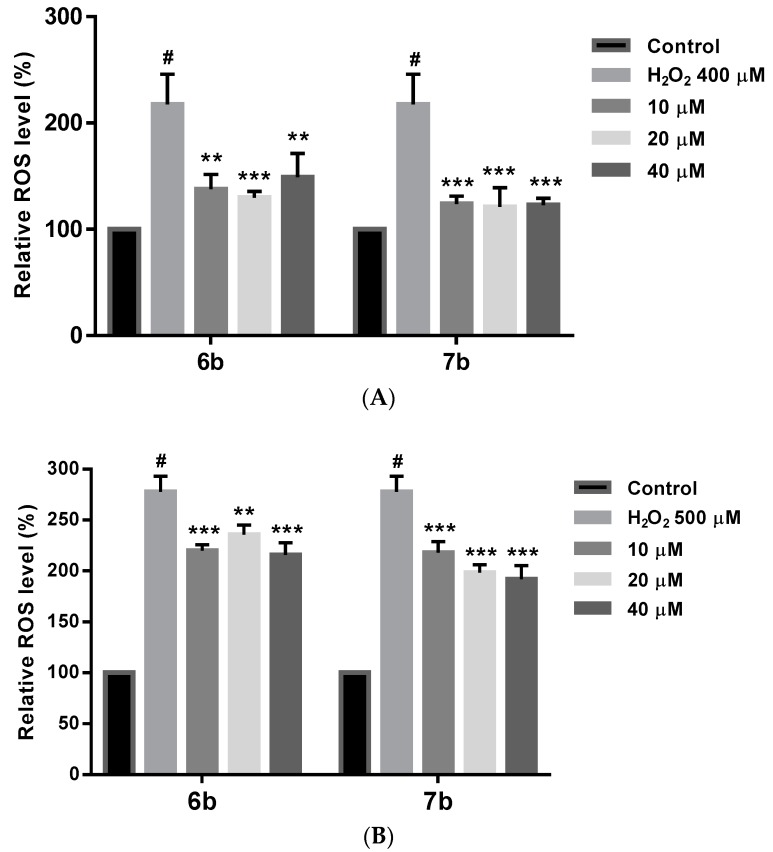
(**A**) Intracellular ROS level reduction by compounds **6b** and **7b** in SH-SH5Y under 4 h treatment with 400 µM H_2_O_2_. Cells were treated with **6b** and **7b** for 24 h prior exposure to H_2_O_2_. ROS levels were evaluated by the DFCH-DA method. (**B**) Same results for **6b** and **7b** in SH-SH5Y under 2 h treatment with 500 µM H_2_O_2_. Cells were treated with **6b** and **7b** for 6 h prior exposure to H_2_O_2_. ROS levels were evaluated by the DFCH-DA method. Data are presented as Mean ± SD of three independent experiments carried out by quadruplicate. ^#^ *p* < 0.001 vs. vehicle-treated control. * *p* < 0.05, ** *p* < 0.01, *** *p* < 0.001 vs. only H_2_O_2_ treated cells.

Oxidative stress increases ROS levels, which may cause cell damage and death. Therefore, ROS production caused by H_2_O_2_ was evaluated in cells pretreated with or without derivatives **6b** and **7b**. In these studies, DFCH-DA method was used to measure by fluorescence the ROS intracellular levels [110], under two different compound treatment regimens. In the first assay, (Figure 12A) a 24 h compound treatment prior to 400 µM H_2_O_2_ exposure for 4 h, afforded a significant ROS level reduction at all the doses assayed, being more significant that reduction for **7b** (*p* < 0.001). In a second study (Figure 12B) a 6 h treatment with **6b** and **7b** followed by 2 h 500 µM H_2_O_2_ exposure has also shown a significant ROS level decrease after compounds treatment. From both experiments, it could be concluded that **6b** and **7b** reduce intracellular ROS, and that longer treatment period seems to be needed to achieve a better ROS reduction.

### 2.9. Protection against Mitochondrial Membrane Depolarization Induced by Oxidative Stress

Depolarization of the Mitochondrial Membrane Potential (MMP) due to the generation of ROS is proposed to participate in mitochondrial dysfunction and cellular apoptosis.

**Figure 13:**
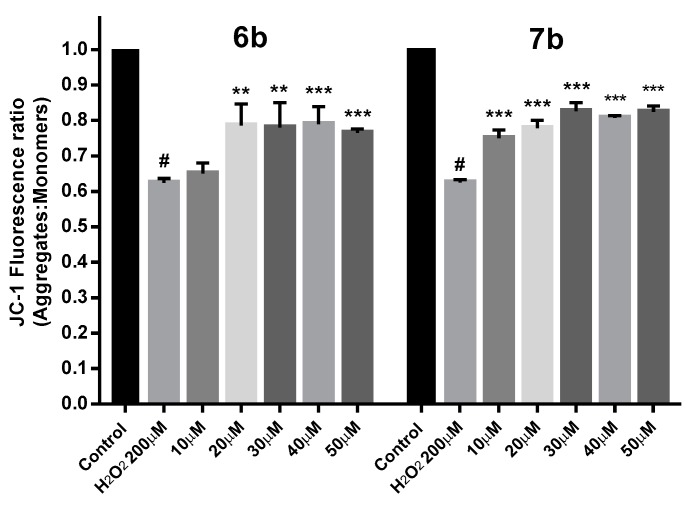
MMP loss is prevented by compounds **6b** and **7b** in SH-SH5Y under 24 h exposure with 200 µM H_2_O_2_. Cells were treated with **6b** and **7b** for 24 h prior exposure to H_2_O_2_. MMP is expressed as JC-1 fluorescence ratio in terms of red fluorescence to green fluorescence (Aggregates:Monomers). Data are presented as Mean ± SD of five independent experiments carried out by quadruplicate. ^#^ *p* < 0.001 vs. vehicle-treated control. * *p* < 0.05, ** *p* < 0.01, *** *p* < 0.001 vs. only H_2_O_2_ treated cells.

To know whether β-diketones **6b** and **7b** will protect the mitochondrial membrane from depolarization under oxidative stress conditions, MMP experiments were carried out with the cationic dye JC-1 (5,5′,6,6′-tetrachloro-1,1′,3,3′-tetraethylbenzimi-dazolylcarbocyanine iodide). JC-1 enters selectively into mitochondria and reversibly change color from red to green as the membrane potential decrease [111]. In healthy cells, JC-1 forms red fluorescent J-aggregates while in apoptotic cells with low MMP, JC-1 remains as monomers with green fluorescence. The ratio of J-aggregates to J-monomers is an indication of healthy non-apoptotic cells. As Figure 13 shows, in SH-SY5Y cells exposed to 200 µM H_2_O_2_ for 24 h, the MMP was reduced about 62%, but those previously treated with **6b** and **7b** at different concentrations for 24 h has shown an MMP recovery of 18–25%. These results confirmed that the neuroprotective effect of **6b** and **7b** is in line with the reduction of intracellular ROS levels and the MMP recovery under H_2_O_2_ oxidative stress insult.

**Figure 14:**
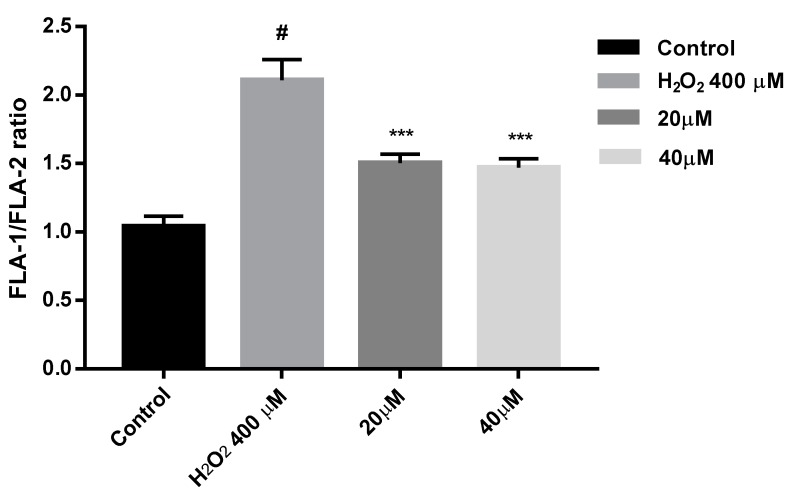
MMP loss is prevented by compound **7b** in SH-SH5Y under 4 h exposure with 400 µM H_2_O_2_. Cells were treated with **7b** for 24 h prior exposure to H_2_O_2_. MMP is expressed as JC-1 fluorescence ratio in terms of red fluorescence to green fluorescence (FLA-1:FLA-2). Data are presented as Mean ± SD of four independent experiments carried out in triplicate. Comparisons between control and treatment groups were made using one-way ANOVA. ^#^ *p* < 0.001 vs. vehicle-treated control. *** *p* < 0.001 vs. only H_2_O_2_ treated cells.

To confirm this MMP recovery results, flow cytometry experiments were carried out with compound **7b** and SH-SY5Y cells. Cells were treated with **7b** for 24 h and then were exposed to 400 µM H_2_O_2_ for 4 h. JC-1 was used as fluorescence indicator of MMP state. In flow cytometry studies of MMP, JC-1 is excited at 488 nm wavelength. Both JC-1 aggregates and monomers show green fluorescence (peak emission at 527 nm) measured in the FL1 channel. JC-1 aggregates exhibit red fluorescence (peak emission at 590 nm) measured in the FL2 channel. Thus, healthy non-apoptotic cells are detected in both FL1 and FL2 channels, while cells with disrupted mitochondrial function show fluorescence in FL1 channel but reduced FL2 intensity [112]. Results obtained from this study (Figure 14) show that cells under oxidative stress induced by 400 µM H_2_O_2_, presented a 2-fold increase in the FLA-1/FLA-2 ratio, while those previously treated with **7b** and then with 400 µM H_2_O_2_ for 4 h, exhibit a significant reduction of that ratio. From these flow cytometry experiments, we can confirm that after compound **7b** treatment there is a significant MMP recovery in SH-SY5Y cells under oxidative stress pressure. 

## 3. Conclusions

In the present work, we report the synthesis and X-ray structures of new asymmetrical β-diketones, which have also shown interesting antioxidant properties in the ABTS, FRAP and DPPH assays, in parallel with improved drug-like physicochemical properties when they were compared with curcumin. The cytotoxicity assays performed against the SH-SY5Y neuronal cell line have identified compounds **6b** and **7b** as the most promising derivatives for progression to neuroprotection studies. In contrast to well-known issues of curcumin, compounds **6b** and **7b** have also demonstrated good solubility and chemical stability in biorelevant media. As potential iron chelators, both β-diketones **6b** and **7b** have a similar profile to that of curcumin, with a better chelating behavior in aqueous media. In the neuroprotection studies carried out, **6b** and **7b** have showed that they were able to protect neuronal SH-SY5Y cells under oxidative stress conditions, with a reduction of the intracellular ROS levels and a significant recovery of the mitochondrial membrane potential. In summary, this new chemotype with drug-like properties affords the possibility of further chemical exploration, as well as its progression to other in vitro and in vivo studies focused to demonstrate their potential as new therapeutic agents for neurodegenerative diseases.

## 4. Experimental Section

### 4.1. General

All chemicals cited in the synthetic procedures were commercial compounds. Melting points were determined by DSC with a DSC 220 C instrument (SEIKO Instruments Inc., Torrance, CA, USA) connected to a model SSC5200H disk station. Thermograms (sample size 0.003–0.005 g) were recorded with a scan rate of 5.0 °C. Column chromatography was performed on silicagel (Merck 60, 70–230 mesh) and elemental analyses using a model 240 apparatus (PerkinElmer, Waltham, MA, USA).

### 4.2. NMR Parameters

Solution spectra were recorded on a 9.4 Tesla spectrometer (Bruker Española S.A., Madrid, Spain, 400.13 MHz for ^1^H, 376.50 MHz for ^19^F, and 100.62 MHz for ^13^C and 40.54 MHz for ^15^N) at 300 K with a 5-mm inverse detection H**-**X probe equipped with a z**-**gradient coil or with a QNP 5 mm probe (^19^F). Chemical shifts (*δ* in ppm) are given from internal solvents: CDCl_3_ 7.26 for ^1^H and 77.0 for ^13^C, DMSO**-***d*_6_ 2.49 for ^1^H and 39.5 for ^13^C. External references were used for ^15^N and ^19^F, nitromethane and CFCl_3_. CPMAS NMR spectra have been obtained on a 9.4 Tesla spectrometer at 300 K (100.73 MHz for ^13^C) using a 4 mm DVT probehead at spinning rates of 12. ^13^C spectra were originally referenced to a glycine sample and then the chemical shifts were recalculated to the Me_4_Si (for the glycine carbonyl atom *δ* = 176.1 ppm).

Solid-state ^19^F (376.94 MHz) spectra have been obtained using a MAS DVT BL2.5 X/F/H double resonance probehead. Samples were spun at the magic angle at rates of 25 kHz and the experiments were carried out at 300 K. The ^19^F spectra were referenced to ammonium trifluoroacetate sample and then the chemical shifts were recalculated to the CFCl_3_ (*δ*CF_3_CO_2_^−^NH_4_^+^ = −72.0 ppm). 

### 4.3. Preparation of Compounds ***1a***–***7a*** and ***1b***–***7b***

(2*Z*,4*E*)-3-Hydroxy-5-(4-hydroxy-3-methoxyphenyl)-1-phenylpenta-2,4-dien-1-one [**1a**, m.p. 159.9 °C] [77], (2*Z*,4*E*)-5-(4-fluoro-3-methoxyphenyl)-3-hydroxy-1-phenylpenta-2,4-dien-1-one [**2a**, m.p. 127.2 °C] [78], (2*Z*,4*E*)-5-(3-fluoro-4-hydroxyphenyl)-3-hydroxy-1-phenylpenta-2,4-dien-1-one [**3a**, m.p. 193.3 °C] [78], (2*Z*,4*E*)-5-(2-fluoro-4-hydroxyphenyl)-3-hydroxy-1-phenylpenta-2,4-dien-1-one [**4a**, m.p. 164.5 °C] [79], (2*Z*,4*E*)-5-(2,4-difluoro-3-hydroxyphenyl)-3-hydroxy-1-phenylpenta-2,4-dien-1-one [**5a**, m.p. 195.0 °C] [79], (2*Z*,4*E*)-5-(2,5-difluoro-4-hydroxyphenyl)-3-hydroxy-1-phenylpenta-2,4-dien-1-one [**6a**, m.p. 189.8 °C] [79], (3*Z*,5*E*)-1,1,1-trifluoro-4-hydroxy-6-(4-hydroxy-3-methoxyphenyl)hexa-3,5-dien-2-one [**1b**, m.p. 108.9 °C] [78], (3*Z*,5*E*)-1,1,1-trifluoro-6-(4-fluoro-3-methoxyphenyl)-4-hydroxyhexa-3,5-dien-2-one [**2b**, m.p. 110.8 °C] [79], (3*Z*,5*E*)-1,1,1-trifluoro-6-(3-fluoro-4-hydroxyphenyl)-4-hydroxyhexa-3,5-dien-2-one [**3b**, m.p. 134.4 °C] [79], (3*Z*,5*E*)-1,1,1-trifluoro-6-(2-fluoro-4-hydroxyphenyl)-4-hydroxy-hexa-3,5-dien-2-one [**4b**, m.p. 151.2 °C] [79], (3*Z*,5*E*)-6-(2,4-difluoro-3-hydroxyphenyl)-1,1,1-trifluoro-4-hydroxyhexa-3,5-dien-2-one [**5b**, m.p. 129.1 °C] [79], (3*Z*,5*E*)-6-(2,5-difluoro-4-hydroxyphenyl)-1,1,1-trifluoro-4-hydroxyhexa-3,5-dien-2-one [**6b**, m.p. 190.8 °C] [79], were prepared as described in the literature.

**Figure 15:**
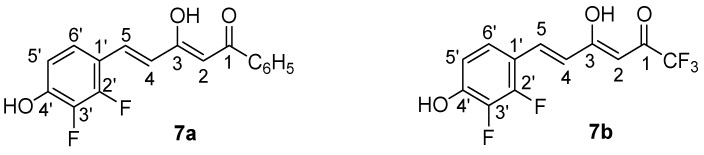
Atom numbering for NMR assignments in compounds **7a** and **7b**.

*(2Z,4E)-5-(2,3-Difluoro-4-hydroxyphenyl)-3-hydroxy-1-phenylpenta-2,4-dien-1-one* (**7a**). 1-Phenylbutane-1,3-dione (2.43 g, 15 mmol) and boric anhydride (731.1 mg, 10.5 mmol) were mixed in dry ethyl acetate (11.25 mL) at 50 °C for 30 min. After that, a mixture of 2,3-difluoro-4-hydroxybenzaldehyde (2.37 g, 15 mmol) and tributyl borate (6.0 mL, 22.5 mmol) in dry ethyl acetate (7.5 mL) was added, with stirring for 30 min at 50 °C. A solution of butylamine (1.5 mL, 15 mmol) in dry ethyl acetate (7.5 mL) was added slowly over 15 min. The reaction mixture was stirred at 50 °C for 90 min and then overnight at room temperature. Hydrochloric acid (1 M, 30 mL) was then added to the solution at 50 °C, and then the system was stirred for 1 h. After cooling, the organic layer was separated from the aqueous layer and extracted with 3 × 15 mL of ethyl acetate. The organic layer was washed with water (2 × 10 mL) and dried (Na_2_SO_4_), and the solvent was evaporated under vacuum. The crude product was crystallized in CHCl_3_/MeOH obtaining the desired product **7a** (Figure 15) as yellow crystals (2.18 g, 48%): m.p. 179.3 °C; ^1^H-NMR (CDCl_3_): *δ* 7.97 (m, 2H, H*o*), 7.68 (d, ^3^*J*_H4_ = 16.0 Hz, 1H, H5), 7.56 (m, 1H, H*p*), 7.49 (m, 2H, H*m*), 7.24 (m, 1H, H6′), 6.84 (ddd, ^3^*J*_H6′_ = 8.9 Hz, ^4^*J*_F3_ = 7.6 Hz, ^5^*J*_F2_ = 2.0 Hz, 1H, H5′), 6.68 (d, ^3^*J*_H5_ = 16.0 Hz, 1H, H4), 6.35 (s, 1H, H2); ^1^H-NMR (DMSO**-***d*_6_): *δ* 16.26 (s, 1H, OH**-**enol), 11.04 (s, 1H, OH-phenol), 8.02 (m, 2H, H*o*), 7.64 (m, 1H, H*p*), 7.61 (d, ^3^*J*_H4_ = 16.0 Hz, 1H, H5), 7.55 (m, 2H, H*m*), 7.44 (ddd, ^3^*J*_H5′_ = 8.8 Hz, ^4^*J*_F2_ = 8.2 Hz, ^5^*J*_F3_ = 2.2 Hz, 1H, H6′), 6.88 (d, ^3^*J*_H5_ = 16.0 Hz, 1H, H4), 6.86 (ddd, ^3^*J*_H6′_ = 8.8 Hz, ^4^*J*_F3_ = 7.7 Hz, ^5^*J*_F2_ = 2.2 Hz, 1H, H5′), 6.79 (s, 1H, H2); ^19^F-NMR (DMSO**-***d*_6_): *δ* −140.2 (dd, ^3^*J*_F2F3_ = 19.8 Hz, ^4^*J*_H6′_ = 8.2 Hz, F2), −161.5 (dd, ^3^*J*_F3F2_ = 19.8 Hz, ^4^*J*_H5′_ = 7.7 Hz, F3); ^13^C-NMR (DMSO**-***d*_6_): *δ* 188.5 (C1), 179.3 (C3), 149.9 (dd, ^1^*J*_F2_ = 251.8 Hz, ^2^*J*_F3_ = 10.7 Hz, C2′), 148.7 (dd, ^2^*J*_F3_ = 9.1 Hz, ^3^*J*_F2_ = 3.4 Hz, C4′), 139.9 (dd, ^1^*J*_F3_ = 243.2 Hz, ^2^*J*_F2_ = 13.9 Hz, C3′), 135.4 (C*i*), 133.1 (C*p*), 131.4 (t, ^3^*J*_F2_ = ^4^*J*_F3_ = 2.8 Hz, C5), 128.9 (C*m*), 127.3 (C*o*), 123.8 (C4), 123.7 (dd, ^3^*J*_F2_ = 7.8 Hz, ^4^*J*_F3_ = 3.6 Hz, C6′), 114.7 (d, ^2^*J*_F2_ = 9.1 Hz, C1′), 113.5 (t, ^3^*J*_F3_ = ^4^*J*_F2_ = 2.5 Hz, C5′), 97.8 (C2); ^13^C-NMR (CPMAS): *δ* 192.1 (C1), 176.1 (C3), 151.7 (C2′), 147.0 (C4′), 139.3 (C3′), 136.2 (C*_i_*), 134.0 (C5), 134.0 (C*_p_*), 127.8 (C*_o_*), 127.8 (C*_m_*), 125.2 (C4), 125.2 (C6′), 116.5 (C1′), 114.0 (C5′), 98.2 (C2); ^19^F-NMR (MAS): *δ* −132.2 (F2), −156.7 (F3). Anal. Calcd for C_17_H_12_F_2_O_3_: C, 67.55; H, 4.00. Found: C, 67.39; H, 3.91%.

*(3Z,5E)-6-(2,3-Difluoro-4-hydroxyphenyl)-1,1,1-trifluoro-4-hydroxyhexa-3,5-dien-2-one* (**7b**). 1,1,1-Trifluoropentane-2,4-dione (1.8 mL, 15 mmol) and boric anhydride (731.1 mg, 10.5 mmol) were mixed in dry ethyl acetate (11.25 mL) at 50 °C for 30 min. After that, a mixture of 2,3-difluoro-4-hydroxybenzaldehyde (2.37 g, 15 mmol) and tributyl borate (4.0 mL, 15 mmol) in dry ethyl acetate (7.5 mL) was added, with stirring for 30 min at 50 °C. A solution of butylamine (1.5 mL, 15 mmol) in dry ethyl acetate (7.5 mL) was added slowly over 15 min. The reaction mixture was stirred at 50 °C for 90 min and then overnight at room temperature. Hydrochloric acid (1 M, 30 mL) was then added to the solution at 50 °C, and the system was stirred for 1.5 h. After cooling, the organic layer was separated from the aqueous layer and extracted with 3 × 15 mL of ethyl acetate. The organic layer was washed with water (2 × 10 mL) and dried (Na_2_SO_4_), and the solvent was evaporated under vacuum. The crude product was purified by column chromatography with dichloromethane as eluent, obtaining two products: the desired β-diketone **7b** (Figure 15), from crystallization in hexane/acetyl acetate, as yellow crystals (882 mg, 20%), and the enamine **8** (1.05 g, 20%) resulting from the addition of butylamine to **7b**. Enamine **8** was characterized by ^1^H-NMR (CDCl_3_): *δ* 11.33 (1H, s, NH), 7.32 (1H, d, ^3^*J*_H4_ = 16.2 Hz, H5), 7.21–7.14 (1H, m, H6′), 6.90 (1H, d, ^3^*J*_H5_ = 16.1 Hz, H4), 6.90–6.80 (1H, m, H5′), 6.16 (1H, bs, OH), 5.69 (1H, s, H2), 3.46 (2H, m, -C*H*_2_-NH-), 1.69 (2H, m, -CH_2_-C*H*_2_-CH_2_-), 1.47 (2H, m, -CH_2_-C*H*_2_-CH_3_), 0.97 (3H, m, CH_3_); ^13^C-NMR (CDCl_3_): *δ* 175.2 (q, ^2^*J*_CF3_ = 32.6 Hz, C1), 166.0 (C3), 150.0 (dd, ^1^*J*_F2_ = 254.5 Hz, ^2^*J*_F3_ = 11.3 Hz, C2′), 146.9 (dd, ^2^*J*_F3_ = 10.8 Hz, ^3^*J*_F2_ = 2.8 Hz, C4′), 140.4 (dd, ^1^*J*_F3_ = 241.6 Hz, ^2^*J*_F2_ = 15.0 Hz, C3′), 132.8 (d, ^3^*J*_F2_ = 3.2 Hz, C5), 123.8 (t, ^3^*J*_F2_ = ^4^*J*_F3_ *=* 3.9 Hz, C6′), 119.9 (d, ^4^*J*_F2_ = 8.3 Hz, C4), 118.0 (d, ^1^*J* = 271.0 Hz, CF_3_), 116.6 (d, ^2^*J*_F2_ = 8.1 Hz, C1′), 112.9 (d, ^3^*J*_F3_ = 3.3 Hz, C5′), 85.2 (C2), 43.8 (-*C*H_2_-NH-), 31.8 (-CH_2_-*C*H_2_-CH_2_-), 19.9 (-CH_2_-*C*H_2_-CH_3_), 13.6 (CH_3_); ^15^N-NMR (CDCl_3_): *δ* −253.9.

As (3*Z*,5*E*)-4-(butylamino)-6-(2,3-difluoro-4-hydroxyphenyl)-1,1,1-trifluorohexa-3,5-dien-2-one **8** needs stronger hydrolysis conditions to yield **7b**, 12 N HCl (2.5 mL) was added and the solution was stirred for 30 min at 50 °C. After cooling, the aqueous layer was extracted with 3 × 3 mL of ethyl acetate. The organic layer was then washed with water (2 × 2 mL) and dried (Na_2_SO_4_), and the solvent was evaporated under vacuum. The yield for the hydrolysis was 90%, and the global yield for the synthesis of **7b** after crystallization in hexane/ethyl acetate was (1.67 g, yield 38%): m.p. 162.7 °C (from hexane/ethyl acetate); ^1^H-NMR (CDCl_3_): *δ* 14.15 (1H, br, OH-enol), 7.75 (1H, d, ^3^*J*_H4_ = 16.0 Hz, H5), 7.24 (1H, ddd, ^3^*J*_H5′_ = 8.9 Hz, ^4^*J*_F2_ = 7.3 Hz, ^5^*J*_F3_ = 2.1 Hz, H6′), 6.85 (1H, ddd, ^3^*J*_H6′_ = 8.9 Hz, ^4^*J*_F3_ = 7.6 Hz, ^5^*J*_F2_ = 2.0 Hz, H5′), 6.60 (1H, d, ^3^*J*_H5_ = 16.0 Hz, H4), 6.02 (1H, s, H2), 5.59 (1H, bs, OH-phenol); ^1^H-NMR (0.7 CDCl_3_ + 0.1 DMSO-*d*_6_): *δ* 10.41 (1H, s, OH), 7.67 (1H, d, ^3^*J*_H4′_ = 15.9 Hz, H5), 7.10 (1H, ddd, ^3^*J*_H5′_ = 9.5 Hz, ^4^*J*_F2_ = 7.7 Hz, ^5^*J*_F3_ = 2.2 Hz, H6′), 6.71 (1H, ddd, ^3^*J*_H6′_ = 9.2 Hz, ^4^*J*_F3_ = 7.7 Hz, ^5^*J*_F2_ = 1.9 Hz, H5′), 6.51 (1H, d, ^3^*J*_H5′_ = 15.9 Hz, H4), 5.96 (1H, s, H2); ^13^C-NMR (0.7 CDCl_3_ + 0.1 DMSO-*d_6_*): *δ* 180.6 (C3), 179.3 (d, ^2^*J*_CF3_ = 36.1 Hz, C1), 150.3 (dd, ^1^*J*_F2_ = 254.9 Hz, ^2^*J*_F3_ = 10.9 Hz, C2′), 149.3 (dd, ^2^*J*_F3_ = 9.7 Hz, ^3^*J*_F2_ = 3.4 Hz, C4′), 140.0 (^1^*J*_F3_ = 244.4 Hz, ^2^*J*_F2_ = 13.9 Hz, C3′), 135.7 (t, ^3^*J*_F2_ = ^4^*J*_F3_ = 2.8 Hz, C5), 123.4 (t, ^3^*J*_F2_ = ^4^*J*_F3_ *=* 3.8 Hz, C6′), 120.3 (d, ^4^*J*_F2_ = 7.5 Hz, C4), 116.1 (d, ^1^*J* = 285.7 Hz, CF_3_), 114.1 (d, ^2^*J*_F2_ = 8.9 Hz, C1′), 113.0 (t, ^3^*J*_F3_ *=* ^4^*J*_F2_ *=* 2.5 Hz, C5′), 95.0 (d, ^3^*J*_CF3_ = 1.9 Hz, C2); ^19^F-NMR (0.7 CDCl_3_ + 0.1 DMSO-*d*_6_): *δ* −77.6 (CF_3_), −138.4 (dd, ^3^*J*_FF_ = 19.0 Hz, ^4^*J*_H6_ = 7.7 Hz, F2′), −161.3 (ddd, ^3^*J*_FF_ = 19.0 Hz, ^4^*J*_H5_ = 7.7 Hz, ^5^*J*_H6_ = 2.2 Hz, F3′); ^13^C-NMR (CPMAS): *δ* 182.6 (C1 and C3), 151.0 (C2′ and C4′), 141.4 (C3′), 136.6 (C5), 120.8 (C6′), 118.4 (CF3 and C4), 114.2 (C1′ and C5′), 96.3 (C2); ^19^F-NMR (MAS): *δ* −74.2 (CF3), −133.9 (F2), −159.3 (F3). Anal. Calc. for C_12_H_7_F_5_O_3_: C, 49.0; H, 2.4. Found: C, 49.0; H, 2.35%.

### 4.4. X-ray Data Collection and Structure Refinement

Data collection for **7a** and **7b** was carried out at room temperature on a Bruker Smart CCD diffractometer (Bruker Española S.A., Madrid, Spain) using in all cases graphite-monochromated Mo-Kα radiation (λ = 0.71073 Å) operating at 50 kV and 35 mA for **7a** and **7b**. The exposure times were 20 s for **7a** and **7b** in omega.

A summary of the fundamental crystal and refinement data is given in Table 8. The structures were solved by direct methods and refined by full-matrix least-squares procedures on F^2^ (SHELXL-97) [81].

All non-hydrogen atoms were refined anisotropically. The hydrogen atoms were included in their calculated positions and refined riding on the respective carbon atoms with the exception of hydrogens H3A and H2 bonded to O3 and O2 respectively, for **7a** and **7b** that were located in a Fourier synthesis and refined riding on the respective bonded atoms.

CCDC 1561929–1561930 contains the Supplementary crystallographic data for this paper. These data can be obtained free of charge from The Cambridge Crystallographic Data Centre via www.ccdc.cam.ac.uk/data_request/cif. 

### 4.5. Radical Scavenger Capacity Determination

#### 4.5.1. ABTS Assay

ABTS^+^ radical was produced by reacting 2,2′-azino-bis-3-ethylbenzthiazoline-6-sulphonic acid (ABTS) 7 mM with K_2_S_2_O_8_ 2.45 mM, both reactants dissolved in water, and at the volume ratio of 1:1. The mixture was stored in the dark at room temperature for 16 h. The ABTS^+^ solution was diluted to give an absorbance of 0.750 ± 0.025 at 734 nm in EtOH. All the compounds tested were dissolved in EtOH. 150 µL of each compound solution was added to the wells of the 96-well plate, followed by the addition of 50 µL of the ABTS^+^ radical solution. Final compound concentrations tested were 0, 5, 10, 20, 50, 100, 150, 200 µM. The absorbance change at 734 nm was recorded after 30 min incubation in the dark, and the percentage of radical scavenging was calculated for each concentration relative to a blank containing no scavenger. The degree of decolorization was calculated as the percentage reduction of absorbance. The ABTS^+^ radical scavenging activity was calculated as follows:

Percentage ABTS^+^ radical scavenging activity = {1 − [As/Ac]} × 100 (1)

where As is the absorbance of the ABTS^+^ radical solution containing samples and Ac the absorbance of the control solution without antioxidant. The percentages of ABTS^+^ radical reduced were plotted against compound concentration. Trolox was used as a reference antioxidant. All the assays were carried out three times by quadruplicate for each compound.

#### 4.5.2. FRAP (Ferric Reducing Antioxidant Power) Assay

The assay was carried out according to the method of Benzie et al. [86] with slight modifications, in a 96-well microplate. FRAP reagent was prepared by mixing 10 mL of 300 mM acetate buffer with 1 mL of 10 mM 2,4,6-tripyridyl-S-triazine (TPTZ) in HCl 40 mM and 1mL of FeCl_3_·6H_2_O 20 mM. Firstly, 190 µL of the FRAP reagent were added to all wells of the 96-well plate, followed by the addition to the well of 10 µL of the test compounds dissolved in Ethanol to reach the final concentrations assayed (0, 5, 10, 20, 50, 100, 150, 200 µM). After 30 min incubation in the dark, the absorbance was read at 593 nm. Trolox was used as assay control. Three different assays were carried out by quadruplicate for each compound.

#### 4.5.3. DPPH Assay

The assay was performed in a 96-well microplate. A 0.2 mM solution of 2,2-diphenyl-1-picryl-hydrazyl-hydrate (DPPH) in ethanol was prepared. All the compounds tested were dissolved in EtOH. Firstly, 150 µL of each compound solution was added to the wells of the 96-well plate, followed by the addition of 50 µL of the DPPH solution. Final compound concentrations tested were 0, 5, 10, 20, 50, 100, 150, 200 µM. The change in absorbance at 517 nm was measured after 30 min incubation in the dark. The free radical scavenging activity was calculated as inhibition using the following Equation (2):

Percentage DPPH radical scavenging activity = {1 − [As/Ac]} × 100(2)

where As is the absorbance of the DPPH solution containing samples and Ac the absorbance of the control solution without antioxidant but with DPPH. The percentages of DPPH reduced were plotted against compound concentration. The experiment was also conducted using Trolox as reference antioxidant. Assays were performed three times by quadruplicate for each compound.

### 4.6. Cell Culture

Human neuroblastoma SH-SY5Y cells were obtained from Prof. Ricardo Martinez Murillo (Neurovascular Research Group, Department of Translational Neurobiology, Cajal Institute, Madrid, Spain), and cultured in DMEM-F12 (1:1) medium supplemented with 10% heat-inactivated fetal bovine serum (FBS), 1% 100 mg/mL penicillin and 100 mg/mL streptomycin. All cell lines were incubated at 37 °C in a humidified atmosphere incubator of 5% CO_2_. The culture medium was changed every other day, and the cells were sub-cultured after they reached 75–80% confluence.

### 4.7. Cytotoxicity Activity In Vitro in SH-SY5Y Cell Line

SH-SY5Y cells were seeded into 96-well plates at a density of 5 × 10^3^ cells/well and grown in the incubator for 24 h. All compounds tested were dissolved and eventually further diluted in dimethylsulfoxide (DMSO). After overnight incubation, the cells were treated with different test concentrations (5, 10, 20, 50, 100, 150, 200 µM) in a final volume of 200 µL with four replicates each. The concentration of DMSO did not exceed 0.2%, which is considered non-toxic to cells. Cell proliferation or viability was measured using the 3-(4,5-dimethylthiazol-2-yl)-2,5-diphenyl tetrazolium bromide (MTT) assay. After 24 h, 20 µL of MTT (5 mg/mL) was added to each well and the plate was incubated at 37 °C in the dark for 2 h. Supernatants were removed and the formazan crystals were solubilized in DMSO (200 µL/well), under 10 min shaking at room temperature. The reduction of MTT was quantified by absorbance at 570 nm Varioskan microplate reader (Thermo Fisher Scientific, Vantaa, Finland). Effects of the test compounds on cell viability were calculated using cells treated with vehicle as control. The data were subjected to linear regression analysis and the regression lines were plotted for the best fit. The IC_50_ (inhibition of cell viability) concentrations were calculated using the respective dose-response curves. 

### 4.8. SH-SY5Y Cell Neuroprotection Assay

Compound neuroprotection under H_2_O_2_-induced oxidative stress was assessed by MTT assay. Briefly, SH-SY5Y were seeded into 96-well plates at a density of 3 × 10^3^ cells/well and grown in the incubator for 24 h. Cells were pretreated for 24 h with different compound concentrations (5, 10, 20, 30, 40, 50 µM). After the treatment period, cells were exposed to 200 µM H_2_O_2_ for 24 h. Cell viability was measured with MTT following the same procedure described above. Cell viability was expressed as the percentage of the control value (cells without treatment with H_2_O_2_).

### 4.9. Lactate Dehydrogenase (LDH) Cell Viability Assay

SH-SY5Y 96-well plates at a density of 5 × 10^3^ cells/well and grown in the incubator for 24 h. Cells were pretreated for 24 h with different compound concentrations (5, 10, 20, 30, 40, 50 µM) and then exposed to 200 µM H_2_O_2_ for 24 h. Then, the supernatant was used in the LDH assay and the absorbance measured at 490nm in a Varioskan microplate reader after 30 min, according to the manufacturer’s instructions (LDH Cytotoxicity Assay Kit, Pierce^TM^, Thermo Fisher Scientific, Vantaa, Finland). LDH leakage was calculated as the percentage of the control group.

### 4.10. Kinetic Solubility by UV-Spectrometry Assay

A modified protocol was used from that described by Hoelke et al. [99] for the determination of the kinetic solubility from DMSO compound solutions. A compound calibration curve (compound concentration vs. UV absorption) was prepared on a 48 well plate for each compound with concentrations in the range of 10 µM to 1 mM. Each compound dilution was carried out by pipetting defined volumes of a 20 mM stock compound solution in DMSO into the wells and adding PBS 10 mM to reach a final volume of 500 µL/well. The final amount of DMSO per well was 2%. For the lower compound concentrations, an additional volume of DMSO was added into each well to reach the 2% of DMSO. Then, 200 µL of each dilution was transferred to a new 96 well plate for the measurement of the UV-spectra. One well in each row was left without compound as a blank and its UV absorbance was used as an offset correction. The experiments were carried out by duplicate.

In parallel compound saturation solutions were obtained and filtered from 20 mM compound stock in DMSO. So, 10 µL of 20 mM stock solution were dispensed to 190 µL of PBS 10 mM. The solutions were mixed, shaken in a microplate shaker for two hours, and then 160 µL of each solution were filtered and transferred to a new well plate for UV absorption measure. The compound stability in PBS 10 mM was determined at 24, 48 and 72 h with solutions prepared as those for the compound calibration curve.

Measurement of the UV-spectra was performed using a Varioskan microplate reader and spectra were taken in the range of 270–600 nm. Spectral bandwidth was set to 5 nm and the data pint interval was 10 nm. The experiments were carried out by triplicate.

### 4.11. Iron Chelation Capacity

The iron chelation capacity was determined according to the method of Dinis et al. [108], using ferrous chloride instead of ferrous sulfate. In this method, ferrozine can form complexes with a ferrous ion in a quantitative way yielding a red color with maximum absorbance at 562 nm. In the presence of other iron chelators, a decoloration is observed due to the competition with ferrozine for the metal. The assay was carried out in a 96 well plate. Serial dilutions of test compound (0, 5, 10, 25, 50, 100, 150, 200 µM) were dispensed (180 µL) into each well and then 10 µL of FeCl_2_ 1 mM were added (final FeCl_2_ concentration, 50 mM). After shaking the plate for 1 min, 10 µL of Ferrozine 2 mM were added. The mixture was shaken thoroughly and left to stand for 20 min at room temperature. The absorbance was measured at 562 nm with a Varioskan microplate reader. The lower absorbance of the mixture indicates higher chelating activity of the test compound. The compound iron chelation activity was calculated as the percentage of inhibition of the Fe^+2^-Ferrozine complex by the formula in Equation (3):

%Inhibition = {(Ao − As)/Ao} × 100(3)

where Ao is the absorbance of the control, and As is the absorbance in the presence of the test compound. EDTA was used as a positive control. All the experiments were performed three times by quadruplicate for each compound concentration.

### 4.12. Estimation of Intracellular Reactive Oxygen Species (ROS)

Intracellular ROS levels were determined using dichlorofluorescein diacetate (DCFH2-DA) according to Wang et al. [110]. DCFH2-DA is cleaved by intracellular esterases and oxidized by ROS to form the fluorescent compound DCF, which measure represents the ROS level. So, 3 × 10^3^ SH-SY5Y cells were seeded in 96 well plates and after 24 h, treated with the test compound for an additional 24 h. Then, they were exposed to H_2_O_2_ for 24 h. After the incubation period, cells were washed twice with Hanks buffer and loaded with 20 µM DCFH2-DA (in FBS-free culture medium) for 40 min in dark at 37 °C in a CO_2_ incubator. At the end of the dye incubation period, they were washed twice with Hanks buffer to remove the extracellular DCFH2-DA. Fluorescence was measured with a Varioskan microplate reader at 485 nm excitation and 535 nm emission wavelength. Results are expressed as the percentage of fluorescent increase compared with that of the control. All the experiments were performed at least three times by quadruplicate for each compound concentration.

### 4.13. Determination of Mitochondrial Membrane Potential ΔΨ_m_ (MMP)

The fluorescent dye 5,5′,6,6′-tetrachloro-1,1′,3,3′-tetraethylbenzimidazolylcarbocyanine iodide (JC-1) was used for measuring MMP [111]. A decrease in fluorescence intensity represents mitochondrial membrane depolarization. For fluorescence ratio detection under oxidative stress conditions, 96 well plates were seeded with 3 × 10^3^ SH-SY5Y cells and after 24 h incubation they were treated with test compound and left out for 24 h. Then, cells were exposed to different H_2_O_2_ concentrations for 24 h. After the incubation period, cells were washed twice with Hanks buffer and then stained with 5 µM JC-1 in culture media in dark, in a CO_2_ incubator at 37 °C for 20 min. At the end of the dye incubation period, cells were washed three times with Hanks buffer and 200 µL of fresh buffer was added. Cells were analyzed with a Varioskan microplate reader. J-aggregates red fluorescence intensity was measured at 535 nm excitation and 595 nm emission wavelength, while excitation at 485 nm and emission 535 nm wavelength was used for J-monomers green fluorescence. Results were expressed as the ratio of fluorescence intensity of J-aggregates to J-monomers. All the experiments were performed three times by quadruplicate for each compound concentration. In flow cytometry studies of MMP, JC-1 is excited at 488 nm wavelength. Both JC-1 aggregates and monomers exhibit green fluorescence (peak emission at 527 nm) measured in the FL1 channel. JC-1 aggregates show red fluorescence (peak emission at 590 nm) measured in the FL2 channel. Thus, healthy non-apoptotic cells are detected in both FL1 and FL2 channels, while cells with disrupted mitochondrial function show fluorescence in FL1 channel but reduced FL2 intensity.

For MMP flow cytometry studies [112], 10^5^ SH-SY5Y cells were seeded in 6 well plates. After 24 h, cells were treated with test compound and incubated for other 24 h. After the treatment period, cells were exposed to H_2_O_2_ for 4 h. Then, cells were washed twice with Hanks buffer and stained with 5 µM JC-1 in culture media in dark, in a CO_2_ incubator at 37 °C for 20 min. After the staining period, cells were washed three times with Hanks buffer, collected and resuspended in 500 µL culture media. Data were obtained and analyzed using an Accuri C6 flow cytometer (BD Biosciences, Ann Arbour, MI, USA). Experiments were performed three times in duplicate.

### 4.14. Statistical Analysis

Data are expressed as mean ± SD of three or four experiments carried out by quadruplicate unless otherwise indicated. Comparisons were made between control and treated groups or the entire intragroup using two-way ANOVA and Dunnett’s multiple comparisons test using program GraphPad Prism 7.0 (GraphPad-Software, La Jolla, CA, USA) [113].

## Figures and Tables

**Table 1:** Hydrogen bonds (Å and °) for **7a** and **7b**.

Compound	D-H···A	Symmetry Operations	d(D-H)	d(H···A)	d(D···A)	<DHA
**7a**	O3H3A···O4	#1 − x + 1/2, y − 1/2, z + 3/2	1.09	1.66	2.551(2)	135.3
	O2-H2···O4#1		1.19	1.56	2.720(2)	164.5
**7b**	O2-H2···O1		1.10	1.50	2.583(6)	166.7
	O3-H3A···O4		1.10	1.54	2.557(5)	150.7

**Table 2:** Antioxidant capacity from ABTS and FRAP assays.

ABTS Assay			FRAP Assay (%)		
Compound	IC_50_ (µM)	ABTS-TEAC_100_ *	at 100 µM	at 200 µM	FRAP-TEAC_100_ **
**Trolox**	34.0 ± 2.3	1	53.0	100.0	1
**Curcumin**	22.4 ± 0.9	1.05	47.0	74.4	0.86
**1a**	50.5 ± 2.0	0.88	32.2	55.7	0.59
**2a**	>600	0.21	0.1	0.7	0.00
**3a**	60.2 ± 2.2	0.51	20.5	31.0	0.33
**4a**	107.8 ± 7.6	0.40	9.8	14.3	0.15
**5a**	274.7 ± 35.3	0.23	3.4	5.3	0.05
**6a**	67.2 ± 2.7	0.51	15.5	25.2	0.25
**7a**	79.6 ± 4.3	0.69	14.0	20.7	0.22
**1b**	34.6 ± 2.7	0.73	36.6	61.4	0.64
**2b**	>600	0.23	1.9	3.1	0.03
**3b**	113.3 ± 12.4	0.67	19.7	32.6	0.34
**4b**	216.1 ± 39.5	0.52	4.8	8.5	0.09
**5b**	518.1 ± 94.6	0.32	3.0	4.7	0.05
**6b**	115.5 ± 11	0.67	6.7	11.3	0.15
**7b**	70.9 ± 5.8	0.62	8.8	15.3	0.16

* ABTS-TEAC_100_: from ABTS assay. ** FRAP-TEAC_100_: from FRAP assay.

**Table 3:** DPPH^•^ radical scavenging effect (%).

Compound	IC_50_ (µM) *
**Trolox**	19.0 ± 1.8
**Curcumin**	26.5 ± 1.5
**1a**	42.3 ± 3.4
**1b**	53.8 ± 2.6

* Antioxidant concentration that causes a decrease of the initial DPPH^•^ concentration by 50%.

**Table 4:** Calculated physicochemical properties *.

Compound	MWt	MlogP	logD	NO	TPSA	HBD
**Curcumin**	368.39	2.256	2.778	6	96.2	3
**1a**	296.33	2.938	2.467	4	66.8	2
**2a**	298.32	3.863	3.021	3	46.5	1
**3a**	284.29	3.628	2.813	3	57.5	2
**4a**	284.29	3.628	3.023	3	57.5	2
**5a**	302.28	4.014	2.170	3	57.5	2
**6a**	302.28	4.014	2.885	3	57.5	2
**7a**	302.28	4.014	2.858	3	57.5	2
**1b**	288.23	2.046	0.563	4	66.8	2
**2b**	290.22	2.981	0.945	3	46.5	1
**3b**	276.19	2.725	0.972	3	57.5	2
**4b**	276.19	2.725	1.074	3	57.5	2
**5b**	294.18	3.122	0.974	3	57.5	2
**6b**	294.18	3.122	1.158	3	57.5	2
**7b**	294.18	3.122	1.163	3	57.5	2

* Calculations were performed with MedChem Designer [96]. MlogP: Moriguchi LogP; logD: Distribution coefficient at pH 7.4; NO: Number of Oxygen atoms; TPSA: Total Polar Surface Area; HBD: number of Hydrogen Bonding Donor groups.

**Table 5:** Compound cytotoxicity in SH-SY5Y cells.

Compound	IC_50_ (µM) *
**Curcumin**	48.8 ± 5.1
**1a**	103.8 ± 6.7
**2a**	75.7 ± 5.4
**3a**	98.1 ± 8.3
**4a**	103.5 ± 7.1
**5a**	118.7 ± 8.5
**6a**	134.2 ± 6.6
**7a**	125.6 ± 8.0
**1b**	101.8 ± 5.6
**2b**	88.0 ± 4.5
**3b**	109.0 ± 7.6
**4b**	138.0 ± 6.5
**5b**	153.0 ± 21.0
**6b**	186.8 ± 13.2
**7b**	200.0 ± 17.1

* Values are the mean ± SD of three different experiments performed by quadruplicate.

**Table 6:** Solubility in 10 mM PBS.

Compound	Solubility (µg/mL) *
**6b**	281.0 ± 9.0
**7b**	298.2 ± 7.0
**Curcumin**	24.0 ± 3.0

* Values are the mean ± SD of two experiments carried out by triplicate.

**Table 7:** Compound Stability in 10 mM PBS.

Compound	Remaining Compound (%) *			
	0 h	6 h	24 h	48 h
**6b**	100	96.1 ± 0.5	92.2 ± 1.6	90.4 ± 3.0
**7b**	100	95.7 ± 1.0	90.9 ± 1.2	87.8 ± 2.3
**Curcumin**	100	55.9 ± 10.6	42.9 ± 9.0	38.1 ± 7.4

* Values are the mean ± SD of two experiments carried out by triplicate.

**Table 8:** Crystal and refinement data for **7a** and **7b**.

Crystal Data	7a	7b
CCDC code	1561929	1561930
Empirical formula	C_17_H_12_F_2_O_3_	C_14_H_13_F_5_O_4_S
Formula wt	302.27	372.30
Crystal system	Orthorhombic	Orthorhombic
Space group	*Pna*2_1_	*Pbca*
*a*/Å	21.601(2)	10.4091(9)
*b*/Å	12.181(1)	9.1657(8)
*c*/Å	5.418(6)	34.793(3)
a *α*/°	90	90
b β/°	90	90
γ/°	90	90
*V*/Å^3^	1406.6(7)	3319.5(5)
Z	4	8
D_c_/g/cm^3^	1.408	1.490
μ(Mo-Kα)/mm^−1^	0.113	0.262
F(000)	624	1520
θ range/°	1.89 to 27.00	1.17 to 25.00
Index ranges	−27, −1, −6 to 27, 12, 6	−10, −10, −41 to 12, 10, 40
Reflections collected	12,193	23,645
Unique reflections	3098	2932
[Rint]	[0.0523]	[0.0985]
Completeness to theta (%)	100.0	100.0
Data/restraints/params	3098/1/199	2932/0/217
Goodness-of-fit on F^2^	0.994	0.994
R1 (reflns obsd) [I > 2σ(I)] ^a^	0.0373 (1517)	0.0670 (1158)
ωR2 (all data) ^b^	0.0740	0.2398

^a^ R1 = Σ||F_o_| − |F_c_||/Σ|F_o_|. ^b^ ωR2 = {Σ[ω(F_o_^2^ − F_c_^2^)^2^]/Σ[ω(F_o_^2^)^2^]}.

## Supplementary Materials

The following are available online.
